# Genome-wide association study and candidate gene analysis of alkalinity tolerance in *japonica* rice germplasm at the seedling stage

**DOI:** 10.1186/s12284-019-0285-y

**Published:** 2019-04-11

**Authors:** Ning Li, Hongliang Zheng, Jingnan Cui, Jingguo Wang, Hualong Liu, Jian Sun, Tongtong Liu, Hongwei Zhao, Yongcai Lai, Detang Zou

**Affiliations:** 1 Key Laboratory of Germplasm Enhancement, Physiology and Ecology of Food Crops in Cold Region, Ministry of Education, Northeast Agricultural University, Harbin, 150030 China; 2 Heilongjiang Academy of Agricultural Sciences Postdoctoral Programme, Harbin, 150030 China

**Keywords:** *Japonica* rice, Alkalinity tolerance, Gene, Genome-wide association study (GWAS)

## Abstract

**Background:**

Salinity-alkalinity stress is one of the major factors limiting rice production. The damage caused by alkaline salt stress to rice growth is more severe than that caused by neutral salt stress. At present, the genetic resources (quantitative trait loci (QTLs) and genes) that can be used by rice breeders to improve alkalinity tolerance are limited. Here, we assessed the alkalinity tolerance of rice at the seedling stage and performed a genome-wide association study (GWAS) based on genotypic data including 788,396 single-nucleotide polymorphisms (SNPs) developed by re-sequencing 295 *japonica* rice varieties.

**Results:**

We used the score of alkalinity tolerance (SAT), the concentrations of Na^+^ and K^+^ in the shoots (SNC and SKC, respectively) and the Na^+^/K^+^ ratio of shoots (SNK) as indices to assess alkalinity tolerance at the seedling stage in rice. Based on population structure analysis, the *japonica* rice panel was divided into three subgroups. Linkage disequilibrium (LD) analysis showed that LD decay occurred at 109.77 kb for the whole genome and varied between 13.79 kb and 415.77 kb across the 12 chromosomes, at which point the pairwise squared correlation coefficient (*r*^2^) decreased to half of its maximum value. A total of eight QTLs significantly associated with the SAT, SNC and SNK were identified by genome-wide association mapping. A common QTL associated with the SAT, SNC and SNK on chromosome 3 at the position of 15.0 Mb, which explaining 13.36~13.64% of phenotypic variation, was selected for further analysis. The candidate genes were filtered based on LD decay, Gene Ontology (GO) enrichment, RNA sequencing data, and quantitative real-time PCR (qRT-PCR) analysis. Moreover, sequence analysis revealed one 7-bp insertion/deletion (indel) difference in *LOC_Os03g26210* (*OsIRO3*) between the alkalinity-tolerant and alkalinity-sensitive rice varieties. *OsIRO3* encodes a bHLH-type transcription factor and has been shown to be a negative regulator of the Fe-deficiency response in rice.

**Conclusion:**

Based on these results, *OsIRO3* maybe a novel functional gene associated with alkalinity tolerance in *japonica* rice. This study provides resources for improving alkalinity tolerance in rice, and the functional molecular marker could be verified to breed new rice varieties with alkalinity tolerance via marker-assisted selection (MAS).

**Electronic supplementary material:**

The online version of this article (10.1186/s12284-019-0285-y) contains supplementary material, which is available to authorized users.

## Background

Salinity-alkalinity stress is a major constraint to agricultural food production because it decreases crop yield and restricts the use of agricultural land. This problem is increasing annually due to climate change and poor irrigation management (Qadir et al. 2014). Because rice is one of the most important food crops and widely distributed worldwide, monitoring rice planting areas is necessary to ensure national food security (Ma et al. 2016). Rice growth and yield have been strongly influenced by the deterioration and annual increase in the tillage area of saline-alkaline soils (Takagi et al. 2015). Therefore, studying the salinity-alkalinity tolerance of rice has important practical significance for breeding tolerant rice cultivars and improving saline-alkaline land (Li et al. 2014).

Salinity-alkalinity stress restricts the growth and development of rice at all its growth stages by decreasing nutrient solubility, increasing external osmotic pressure, and disrupting ion imbalance, especially cellular pH stability (Chen et al. 2009). High concentrations of Na^+^ in saline-alkaline soils not only alter the cytoplasmic ion strength necessary for cellular metabolism but also disrupt the homeostasis of other mineral elements such as K^+^. Therefore, reducing the concentration of Na^+^ in the cytoplasm is required for salinity-alkalinity tolerance in rice (Bal and Dutt 1986; Jini and Joseph 2017). Based on the differences of main anions, we can divide the salinity-alkalinity stress into two kinds: salt stress and alkalinity stress (Poljakoff-Mayber and Lerner 1999). Numerous studies have shown that in some respects, alkaline soils dominated by NaHCO_3_ and Na_2_CO_3_ may be more stressful than saline soils containing neutral salts such as NaCl and Na_2_SO_4_ (Tanji 2002; Wang et al. 2008).

Salinity-alkalinity tolerance of rice is a very complex quantitative trait that is genetically controlled by multiple quantitative trait loci (QTLs) (Liang et al. 2015). Recently, a large number of studies on QTL mapping of salt tolerance in rice have been carried out (Senadheera et al. 2000; Lin et al. 2004; Liang et al. 2015; Zheng et al. 2015), and some salt-tolerant genes have been obtained by map-based cloning, such as *SKC1* (Ren et al. 2005) and *DST* (Huang et al. 2009). However, progress in the localization of QTLs for alkalinity tolerance under alkaline salt stress (NaHCO_3_ or Na_2_CO_3_) obviously lags behind that for salt tolerance. Moreover, most of the related research is in the primary stage of QTL mapping. Cheng et al. (2008) used ten traits to characterize the alkalinity tolerance of rice at the germination and early seedling stages under a treatment of 0.15% Na_2_CO_3_ alkaline solution, and 14 QTLs controlling the tolerance of alkalinity stress were mapped. The dead leaf rate (DLR) and the dead seedling rate (DSR) were identified using 200 F_2:3_ individuals, thirteen and six QTLs associated with DLR and DSR were detected under alkalinity stress (Qi et al. 2008). A few valuable genes have been found that can be used to improve the alkalinity tolerance of rice. Guo et al. (2014) cloned a rice alkalinity-tolerance gene (*ALT1*) using a mutant that negatively regulates the alkalinity tolerance of rice by preventing oxidative damage. However, *ALT1* also affects root growth during rice seedling development and reduces the number of tillers. Therefore, genetic resources (QTLs and genes) that can be used by rice breeders to improve alkalinity tolerance are needed.

Genome-wide association study (GWAS) is a powerful approach for gaining insight into the genetic architecture of complex traits in many crops and has been used to identify loci and candidate genes (Zhao et al. 2011; Zhou et al. 2015; Huang et al. 2016). Compared to traditional QTL linkage analysis, GWAS is based on high-density variation in natural populations and can detect multiple alleles at the same site (Flint-Garcia et al. 2003). Many QTLs associated with multiple traits have been identified, such as agronomic traits (Huang et al. 2010; Zhao et al. 2011; Yang et al. 2014) and traits associated with abiotic stress (Lv et al. 2015; Pan et al. 2015; Wang et al. 2016; Shakiba et al. 2017). Several loci associated with salt tolerance were also identified in rice based on GWAS. Campbell et al. (2017) found that the genetic basis of root Na^+^ content varied between *indica* accessions and *japonica* accessions via GWAS, and a major QTL (*RNC4*) associated with root Na^+^/K^+^ ratio and root Na^+^ content was identified in a region of approximately 575 kb on chromosome 4. Yu et al. (2017) used 295 rice varieties to perform a GWAS of salt tolerance-related traits in rice at the seedling stage, and 25 SNPs were significantly associated with six phenotypes. Kumar et al. (2015) performed a GWAS of 12 different salt tolerance-related traits in rice and identified 22 SNPs significantly associated with Na^+^/K^+^ ratio and 44 SNPs with other traits observed under salt stress condition. In summary, GWAS is a powerful strategy for mapping QTLs of salt tolerance in rice. However, no studies have dissected the QTLs associated with alkalinity tolerance of rice through GWAS.

In this study, 295 *japonica* rice varieties were collected to evaluate alkalinity tolerance at the seedling stage under alkalinity stress conditions. Eight QTLs significantly associated with the score of alkalinity tolerance (SAT), concentration of Na^+^ in the shoots (SNC) and Na^+^/K^+^ ratio of shoots (SNK) were identified by GWAS. A common QTL on chromosome 3 associated with the SAT, SNC and SNK was selected for further analysis. Finally, *LOC_Os03g26210* was identified as the most likely candidate gene for alkalinity tolerance of *japonica* rice based on linkage disequilibrium (LD) decay, Gene Ontology (GO) enrichment, RNA sequencing data, quantitative real-time PCR (qRT-PCR) and sequence analysis. This result will be useful for improving alkalinity tolerance in *japonica* rice.

## Results

### Phenotypic variation of 295 *japonica* rice varieties in response to alkalinity treatment

**Fig. 1:**
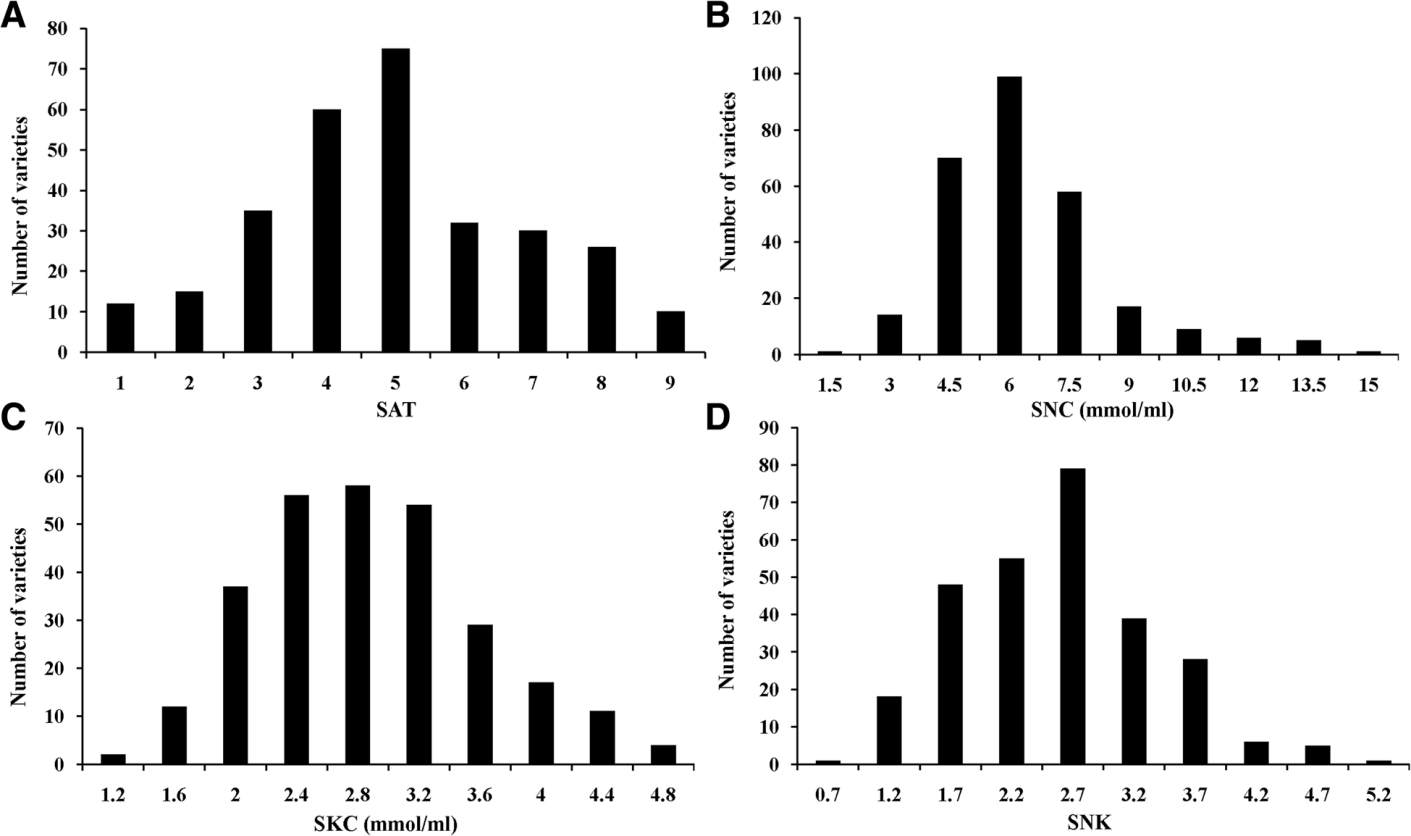
Phenotypic variation in the SNC, SKC, SNK and SAT in 295 *japonica* rice varieties. **a**, The score of alkalinity tolerance (SAT). **b**, The concentration of Na^+^ in the shoots (SNC). **c**, The concentration of K^+^ in the shoots (SNC). **d**, The Na^+^/K^+^ ratio of shoots (SNK)

To assess the phenotypic variation in alkalinity tolerance in 295 *japonica* rice varieties at the seedling stage, four alkalinity tolerance indices were evaluated: the SAT, SNC, concentration of K^+^ in the shoots (SKC) and SNK. Under the alkalinity stress treatment, the average SAT value was 5.02 (Additional file 1: (Table S1). A total of 12 varieties were highly tolerant (SAT = 1) and 10 varieties were highly sensitive (SAT = 9) (Fig. 1). The variation in the SNC and SKC ranged from 1.30 ~ 15.57 mmol/ml and 1.15 ~ 7.74 mmol/ml, respectively, and the coefficient of variation was 35.20% and 27.81%, respectively (Additional file 1: Table S1). The variation in SNK was between 0.64 and 2.04, and the coefficient of variation is 31.08% (Additional file 1: Table S1). Therefore, the SAT, SNC, SKC and SNK of rice seedlings were affected to varying degrees by alkalinity stress, and there were large variations among the 295 *japonica* rice varieties at the seedling stage. The correlation analysis suggested that there was a significant positive correlation between the SNC and SAT (r^2^ = 0.577^**^) and a significant negative correlation between the SNC and SKC, for which the correlation coefficient was − 0.335^**^ (Table 1). Statistical analysis (Fig. 1) revealed continuous variation in each trait, which was consistent with the genetic characteristics of quantitative traits controlled by multiple genes.

**Table 1:** Correlation coefficients between alkalinity tolerance-related traits in 295 *japonica* rice varieties

	SAT	SNC	SKC	SNK
SAT	1			
SNC	0.577^a^	1		
SKC	-0.476	-0.335^a^	1	
SNK	0.339	0.637^a^	-0.399	1

^a^: Indicates significance at the 1 % levels

### SNP validation, population structure and LD analysis

Re-sequencing of the 295 *japonica* rice accessions by an Illumina HiSeq XTen sequencer generated a total of 1798.65G of clean data, with an average depth of 14.62× and a coverage of the Nipponbare reference genome of 96.51%. After mapping against the reference genome, a total of 3,437,749 non-redundant SNPs were identified. Based on the criteria of having less than 20% missing data and a minor allele frequency (MAF) greater than 5% in the selected population, a total of 788,396 SNPs were selected for GWAS.

**Fig. 2:**
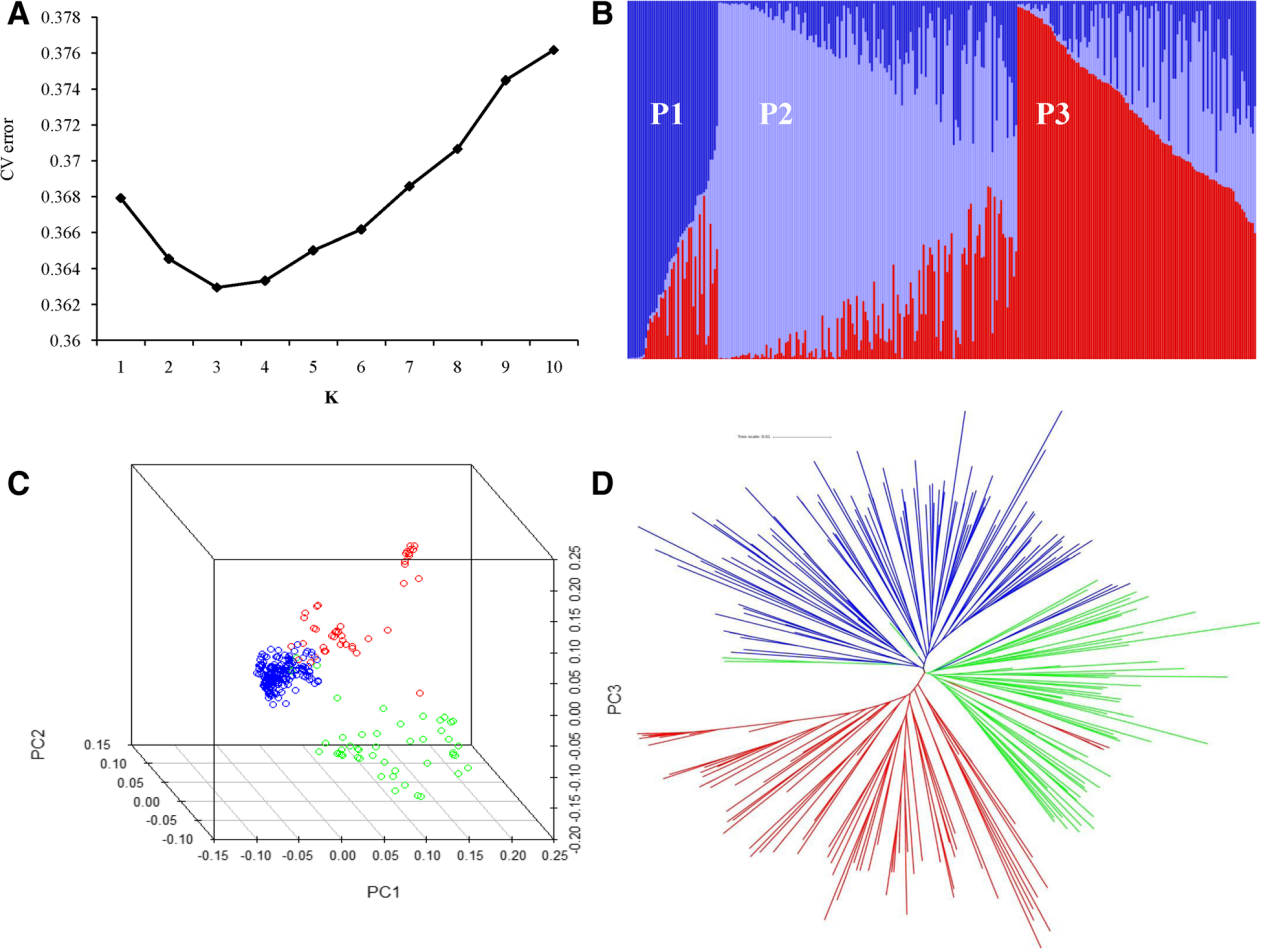
Population structure of 295 *japonica* rice varieties. **a**, The CV error of each K value. **b**, Subgroups (K = 3) inferred using ADMIXTURE software. **c**, Principal component analysis of 295 *japonica* rice varieties. Colors of green, blue, and red represent P1, P2 and P3 in Fig. 2b, respectively. **d**, Neighbor-joining tree of 295 *japonica* rice varieties. Colors of green, blue, and red represent P1, P2 and P3 in Fig. 2b, respectively

We used ADMIXTURE software to calculate the genetic components of each variety. The K value with the lowest cross-validation (CV) error was considered the number of subgroups (Fig. 2a). The 295 *japonica* rice varieties were divided into three subgroups, designated P1, P2 and P3. (Fig. 2b, Additional file 2: Table S2). Based on principal component analysis (PCA), the *japonica* rice panel formed three subgroups with different distributions along the first two eigenvectors; PC1 and PC2 accounted for 19.95 and 12.96% of the genetic variation, respectively (Fig. 2c). Additionally, a neighbour-joining (NJ) tree was constructed based on Nei’s genetic distance with the three clusters (red, blue, and green) (Fig. 2d). The combined results of ADMIXTURE, PCA and the NJ tree divided the *japonica* rice panel into three subgroups, and these varieties did not show an extremely differentiated population structure.

In the whole panel, the *r*^2^ estimate was as high as 0.83 for the whole genome (Additional file 3: Figure S1) and ranged from 0.76 to 0.89 among the individual chromosome (Additional file 4: Figure S2). As expected, the *r*^2^ value declined with increasing physical distance between markers. The average *r*^2^ for the whole genome decreased to half of its maximum value at 109.77 kb. The LD decay was slower for chromosomes 2, 3, 4, 5, 6 and 12. The average *r*^2^ decreased to half of its maximum value at 203.70 kb, 162.55 kb, 118.49 kb, 415.77 kb, 344.51 kb and 186.17 kb, respectively. The LD decay was faster for chromosomes 1, 7, 8, 9, 10 and 11. The average *r*^2^ decreased to half of its maximum value at 24.74 kb, 74.56 kb, 90.14 kb, 52.08 kb, 68.13 kb and 13.79 kb, respectively (Additional file 4: Figure S2). Therefore, the use of these varieties had a slight advantage over that of the other sets of *japonica* rice *germplasm* because it resulted in the inclusion of fewer candidate genes within an LD block (Zhao et al. 2011; Yano et al. 2016).

### GWAS analysis

**Fig. 3:**
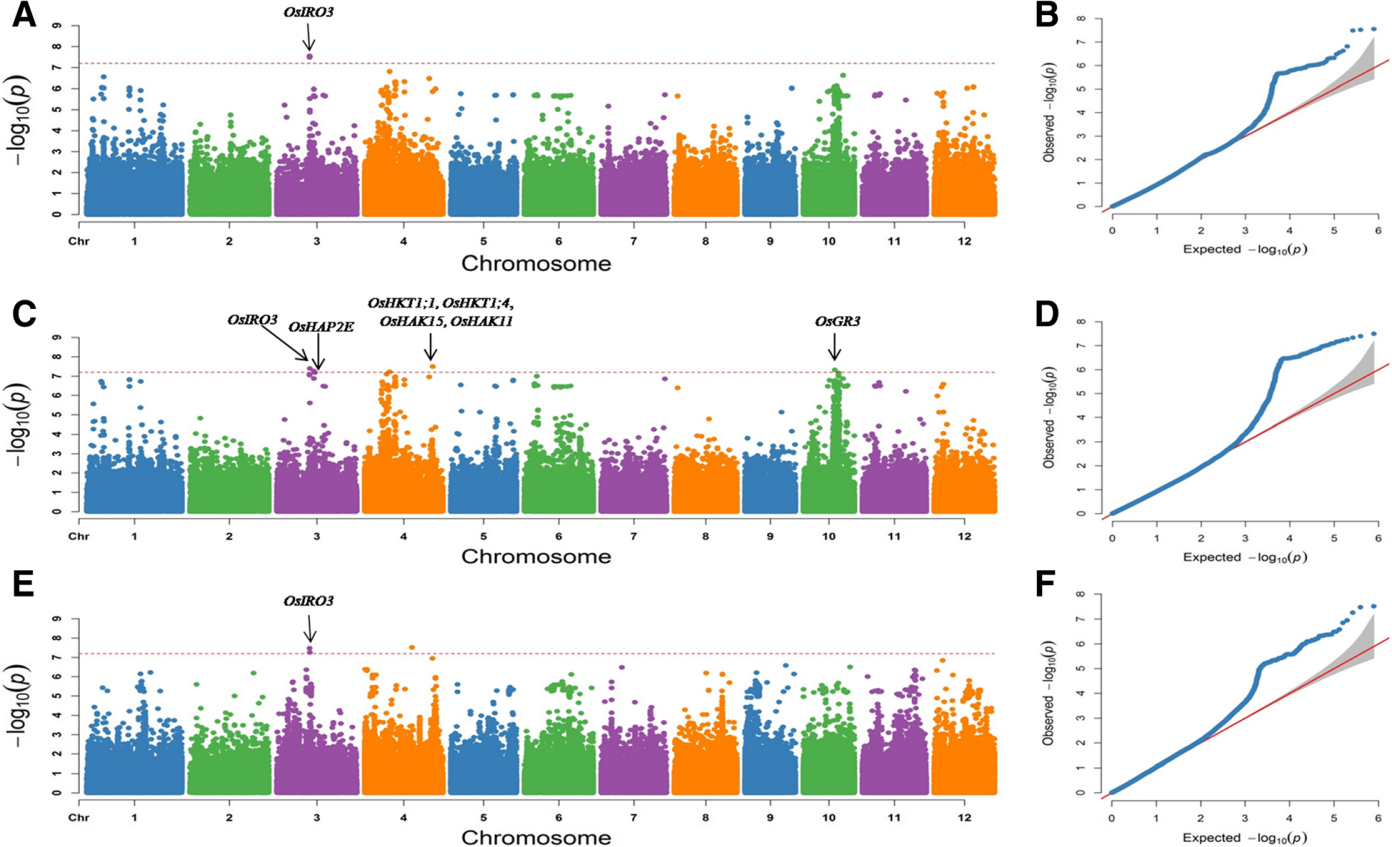
Manhattan plots and quantile-quantile (Q-Q) plots of genome-wide association studies for the SNC, SNK and SAT**. a**, Manhattan plot for the SNC. **b**, Q-Q plot for the SNC. **c**, Manhattan plot for the SNK. **d**, Q-Q plot for SNK. **e**, Manhattan plot for the SAT. **f**, Q-Q plot for the SAT

GWAS was performed via the mixed linear model (MLM) method, considering both population structure (Q) and kinship (K), in Tassel 5.0 software. According to the LD decay rate for the 12 chromosomes in the 295 *japonica* rice accessions (Additional file 4: Figure S2), a region was considered one QTL if it had more than two SNPs with *P* < 6.34E-08 within an LD interval. A Manhattan plot of the GWAS results is shown in Fig. 3. In total, eight QTLs with 11 SNPs were significantly associated with alkalinity tolerance in the 295 *japonica* rice accessions (Table 2). These QTLs were distributed on chromosomes 3, 4, and 10, and the *R*^*2*^ ranged from 13.71% to 13.97%. Three significant SNPs for the SAT were distributed on chromosomes 3 and 4 and contained two genome intervals, named *qSAT3* and *qSAT4,* respectively. Three significant SNPs for the SNC were distributed on chromosome 3 and contained one genome interval, named *qSNC3.* Five significant SNPs for the SNK were distributed on chromosomes 3, 4 and 10 and contained five genome intervals, named *qSNK3–1, qSNK3–2, qSNK4–1, qSNK4–2* and *qSNK10*, respectively (Fig. 3, Table 2). No significant SNPs were detected for the SKC. Among these QTLs, *qSAT3, qSNC3* and *qSNK3–1* were located in the same LD interval near position 15.0 Mb with the lowest *P* value (*P =* 2.87E-08) and were considered one QTL, which explained 13.36~13.64% of the total phenotypic variation.

**Table 2:** The mapped QTLs associated with alkalinity tolerance of *japonica* rice at seedling stage

Traits	QTLs	Lead SNPs	Chr.	Position	*P* value	R^2^(%)	Known genes
SAT	*qSAT3*	Chr3_14978662	3	14978662	3.36E-08	13.36	*OsIRO3*
	*qSAT4*	Chr4_21534673	4	21534673	3.87E-08	13.97	
SNC	*qSNC3*	Chr3_14978662	3	14978662	2.87E-08	13.64	*OsIRO3*
SNK	*qSNK3-1*	Chr3_15093871	3	15093871	4.06E-08	13.39	*OsIRO3*
	*qSNK3-2*	Chr3_16913725	3	16913725	5.55E-08	13.17	*OsHAP2E*
	*qSNK4-1*	Chr4_11383297	4	11383297	5.99E-08	13.79	
	*qSNK4-2*	Chr4_30958528	4	30958528	3.23E-08	13.85	*OsHKT1;1, OsHKT1;4*, *OsHAK15, OsHAK11*
	*qSNK10*	Chr10_14244765	10	14244765	4.74E-08	13.42	*OsGR3*

R^2^ (%): Phenotypic variance explained

A comparison with previous results of salinity-alkalinity tolerance gene mapping or functional characterization indicated that six QTLs co-localized with known characterized genes (Table 2). In these QTLs, We found seven genes known to be involved in salinity-alkalinity tolerance of rice: *OsIRO3* (Zheng et al. 2010), *OsHAP2E* (Alam et al. 2014), *OsHKT1;1* (Wang et al. 2015b), *OsHKT1;4* (Suzuki et al. 2016), *OsHAK15* (Bañuelos et al. 2002), *OsHAK11* (Bañuelos et al. 2002) and *OsGR3* (Wu et al. 2015) (Fig. 3). These genes were located within or near six QTL intervals and were identified for the SNC, SNK and SAT via GWAS.

### Candidate gene analysis

**Fig. 4:**
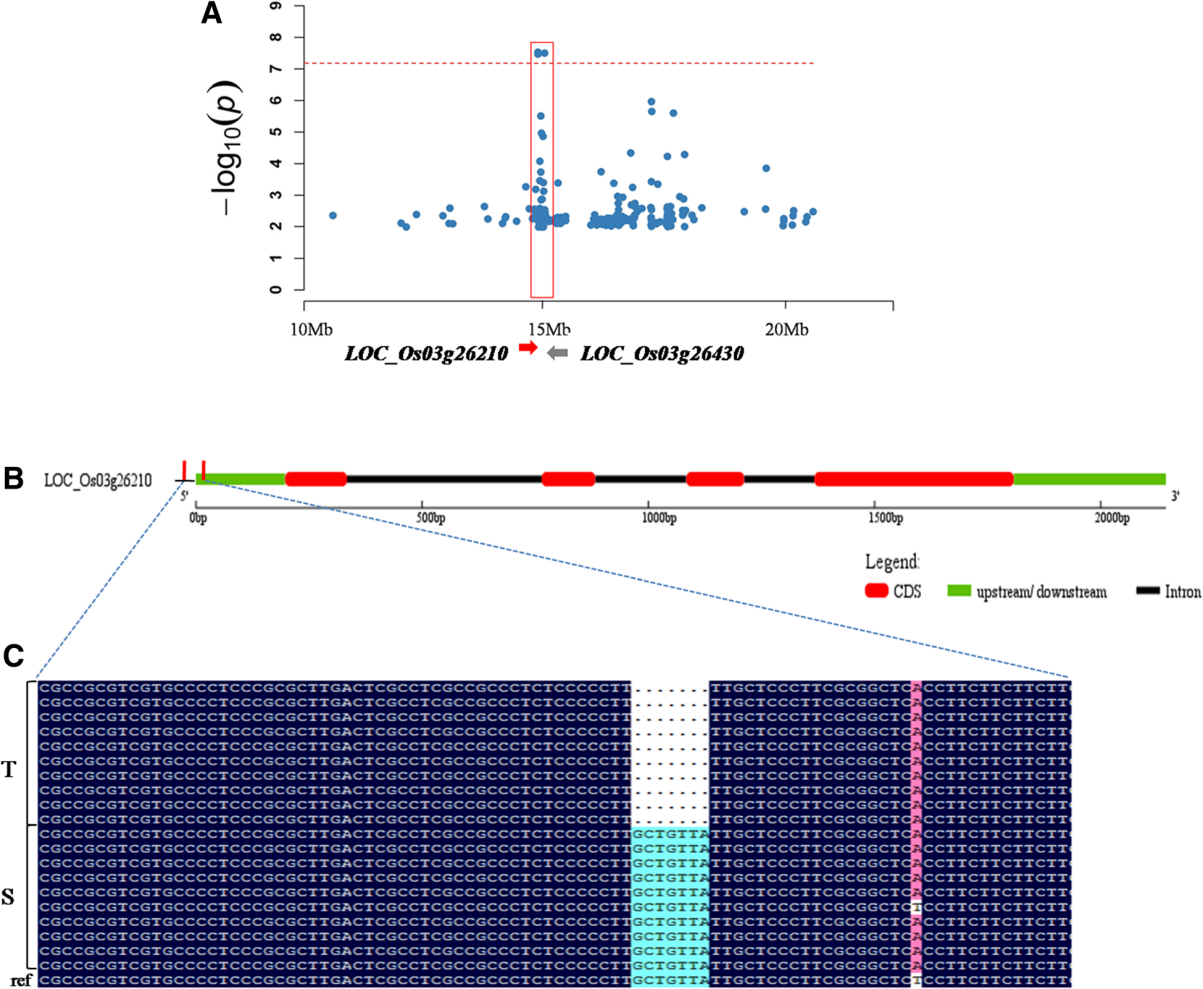
The location of *qSNC3* on chromosome 3 and sequence difference analysis of *LOC_Os03g26210*. **a**, Colocalization of *LOC_Os03g26210* and *LOC_Os03g26430* with *qSNC3*. The arrow indicates the location and direction of *LOC_Os03g26210* and *LOC_Os03g26430*. **b**, The gene structure of *LOC_Os03g26210*. **c**, Sequence differences in *LOC_Os03g26210* between ten alkalinity-tolerant varieties (low SAT) and ten alkalinity-sensitive varieties (high SAT). T indicates alkalinity-tolerant varieties, S indicates alkalinity-sensitive varieties, Ref is the reference sequence of Nipponbare genome

**Fig. 5:**
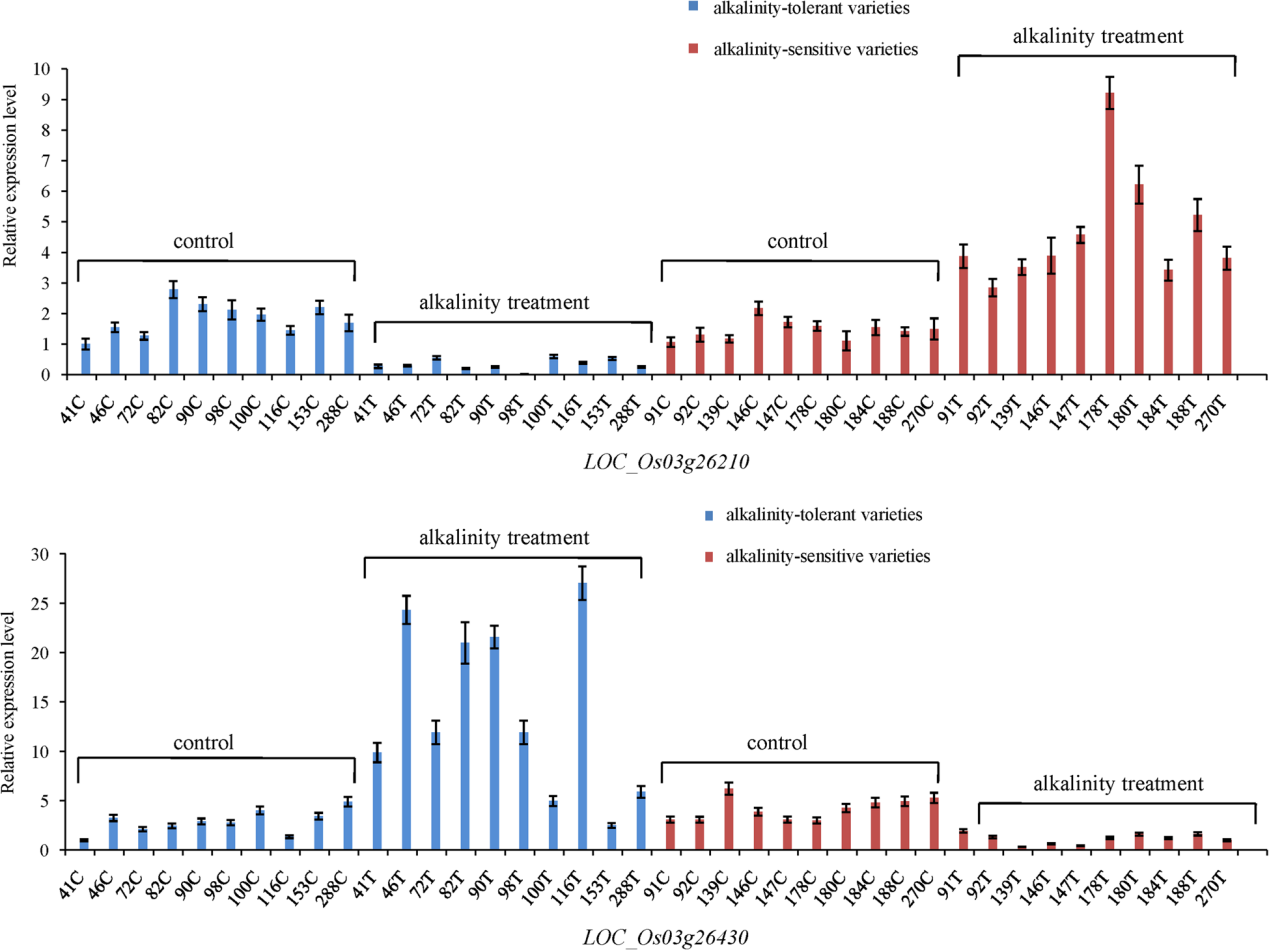
Expression patterns of *LOC_Os03g26210* and *LOC_Os03g26430* under normal growth conditions and alkalinity stress conditions. C indicates normal growth conditions, T indicates alkalinity stress conditions. The number preceding C and T indicates the number of rice varieties

The GWAS analysis detected a common QTL for the SAT, SNC and SNK on chromosome 3, which harboured the highest-peak SNP at approximately 15.0 Mb (Fig. 4a). According to the LD decay analysis of chromosome 3 (Additional file 4: Figure S2), a 325-kb region was identified as the candidate region, which contained 54 genes including 20 functionally annotated genes, 17 expressed proteins with unknown function, 5 hypothetical proteins and 12 retrotransposon proteins (Additional file 5: Table S3). According to the gene function annotation and GO enrichment analysis (Additional file 6: Table S4), we chose genes with functions related to stress response or metabolic process. We also referred to previously reported RNA sequencing data (Additional file 7: Table S5) to analyse the expression pattern of candidate genes under alkaline stress (Li et al. 2018). Through further integrated analysis, we selected 12 candidate genes (Additional file 8: Table S6) to compare expression levels between alkalinity-tolerant and alkalinity-sensitive varieties by qRT-PCR analysis under normal and alkalinity stress conditions. Two genes (*LOC_Os03g26430* and *LOC_Os03g26210*) were differentially expressed between ten alkalinity-tolerant varieties (SAT = 1) and ten alkalinity-sensitive varieties (SAT = 9) (Fig. 5). Under the alkalinity stress treatment, *LOC_Os03g26210* showed higher expression levels in alkalinity-sensitive varieties than in alkalinity-tolerant varieties (Fig. 5). The opposite expression pattern was observed for *LOC_Os03g26430*. The candidate gene *LOC_Os03g26210* is a gene that have been studied and named (*OsIRO3*) in a prior study (Zheng et al. 2010). This gene encodes a bHLH-type transcription factor and has been shown to be a negative regulator of the Fe-deficiency response in rice. *LOC_Os03g26430* is an aldose 1-epimerase gene, and its function has not been preliminary studied.

### Identification of candidate genes responsible for alkalinity tolerance of rice

**Fig. 6:**
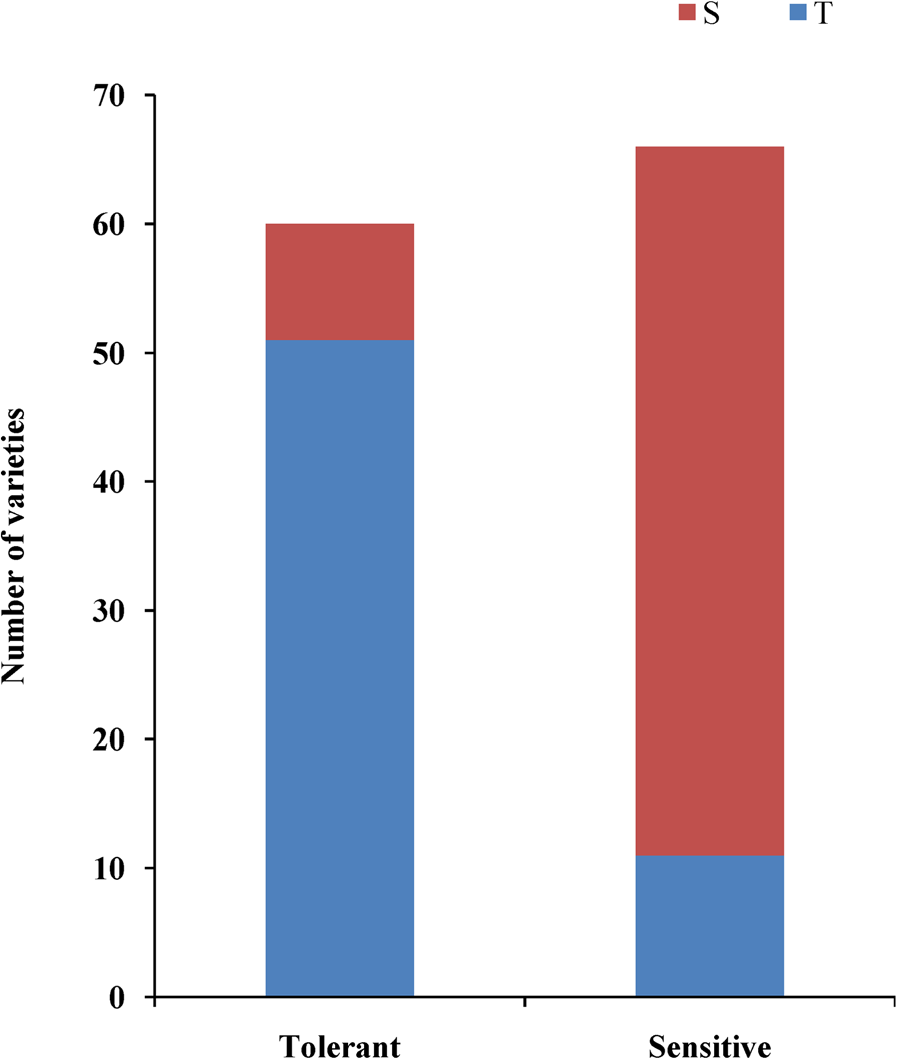
The distribution of tolerant genotype and sensitive genotype in 126 *japonica* rice varieties. T (blue box) indicates tolerant genotype without the 7 bp sequence, S (red box) indicates sensitive genotype with the 7 bp sequence

To identify the association between candidate genes and the alkalinity tolerance phenotype, we sequenced *LOC_Os03g26430* and *OsIRO3* in ten alkalinity-tolerant and ten alkalinity-sensitive varieties. The sequence analysis of *OsIRO3* showed that the alkalinity-tolerant varieties contained one deletion in the starting position of 5′ untranslated region (UTR) (ATG start codon 193–199 bp upstream) with a total length of 7 bp compared with the alkalinity-sensitive varieties (Fig. 4b and c). *LOC_Os03g26430* exhibited no significant sequence differences between alkalinity-tolerant and alkalinity-sensitive varieties. Furthermore, we designed an insertion-deletion (indel) marker using the 7-bp deletion sequence to genotype 126 *japonica* rice varieties (60 tolerant and 66 sensitive varieties). The results showed that 85.00% (51 of 60) of the tolerant varieties (SAT ≤ 3) had the tolerant allele, and 83.33% (55 of 66) of the sensitive varieties (SAT ≥ 6) had the sensitive allele (Fig. 6).

## Discussion

Salinity-alkalinity stress is an important abiotic stress affecting rice cultivation worldwide (Li et al. 2014). In contrast to salinity stress, alkalinity stress can not only cause ionic toxicity in plants but also damage the stability of cells due to its high pH, thus affecting the normal growth of plants (Chen et al. 2009). Phenotypic identification is an important genetic tool for studying alkalinity tolerance in rice. In previous studies, many methods were used to assess alkalinity tolerance of rice, and the determination of concentrations of Na^+^ and K^+^ was one of the commonly methods (Li et al. 2017). Previous studies have shown that alkalinity-tolerant plants can sequester Na^+^ in vacuoles by compartmentalizing of ions at the cellular level to enhance tolerance of a high concentration of ions (Wang et al. 2015a). In this study, there was a significant positive correlation between the SAT and SNC (r^2^ = 0.577**). In addition, excessive accumulation of Na^+^ in the shoots of rice indirectly affects the upward transport of K^+^. This view was supported by the results of this study, which revealed a significant negative correlation between the SNC and SKC (r^2^ = − 0.335**). Moreover, there was a significant positive correlation between the SNC and SNK (r^2^ = 0.637**) (Table 1).

Salinity-alkalinity tolerance is a very complex trait in rice (Zhang et al. 2013). Many genes related to salt tolerance have been identified in rice using multiple strategies. However, few genes have been found to increase the tolerance of rice under alkalinity stress. Therefore, we compared the results identified in this study with the genes related to salt tolerance obtained in previous studies. Six of the eight QTLs co-localized with seven genes found to be involved in salt tolerance of rice in previous studies (Fig. 3, Table 2). *OsHAP2E* was located within *qSNK3–2* and enhance the ability of rice to tolerate high salt and drought stress. *OsGR3* plays an important role in salt tolerance in rice by regulating the glutathione redox state and was found to be located within *qSNK10* in this study. We found similar cases for four genes known to be involved in salt tolerance in rice: *OsHKT1;1, OsHKT1;4*, *OsHAK15* and *OsHAK11*. These genes were located within or near the interval of *qSNK4–2*. Among these genes, *OsHKT1;1* and *OsHKT1;4* are two member of the high-affinity K^+^ transporter (HKT) family and play an important role in reducing the Na^+^ accumulation in shoots to cope with salt stress in rice. *OsHAK15* and *OsHAK11* are located near the lead SNP Chr4_30958528 and involved in the absorption and transport of K^+^ in roots.

Among these QTLs, *qSAT3* overlapped with *qSNC3* and *qSNK3–1*, which harboured the highest-peak SNP on chromosome 3 (at 15.0 Mb), and explained 13.64% of the total phenotypic variation. The LD decay analysis indicated that an approximately 325-kb region at the associated locus was a candidate region for further study (Fig. 4). On the basis of GO enrichment and gene functional annotation results and gene expression profiles before and after alkalinity treatment in rice, we selected 12 candidate genes for gene expression analyses. These methods are commonly used to verify the function of genes identified in GWAS, especially those related to abiotic stress. After filtering using these methods, only two genes remained as candidate genes for the alkalinity tolerance of rice.

Of the two candidate genes, *OsIRO3* contained a 7-bp deletion in the starting position of the 5’UTR that was significantly associated with the alkalinity-tolerant phenotype and thus was the most likely candidate gene, which encodes a rice bHLH-type transcription factor. A previous study indicated that *OsIRO3* is a negative regulator of the Fe-deficiency response in rice (Zheng et al. 2010). Transgenic rice plants over-expressing *OsIRO3* were hypersensitive to Fe deficiency, indicating that the Fe deficiency response was compromised. Furthermore, the Fe concentration in shoots of transgenic rice plants over-expressing *OsIRO3* was less than that in wild-type plants. In this study, *OsIRO3* showed higher expression levels in alkalinity-sensitive varieties than in alkalinity-tolerant varieties. Moreover, the sequence analysis of *OsIRO3* revealed that the tolerant varieties contained one deletion in the starting position of 5’UTR (ATG start codon 193–199 bp upstream) with a total length of 7 bp compared with the sensitive varieties. The 5′ UTR is a piece of RNA or mRNA located at 5′ upstream to the protein-coding region or unit of that RNA or mRNA. This region of mRNA will not be translated into amino acid peptides, but it may play regulatory roles in the translation, transcription and stability of RNA. Therefore, *OsIRO3* not only regulates the Fe-deficiency response in rice but also enhances the alkalinity tolerance of rice by regulating the transcription level of the gene under alkalinity stress.

In addition, we examined the distribution of this 7-bp indel in 66 diverse rice accessions, including six divergent groups of cultivated rice and the wild rice, and have been subjected to pan-genome analysis (Zhao et al. 2018). The results showed that 9 of the 66 rice accessions contained this 7-bp deletion, and these 9 rice accessions were *O. sativa* temperate *japonica*, which were GP551, GP677, GP669, HP13–2, HP390, KY131, LG31, IL9 and UR28. Moreover, this deletion fragment was not detected in other five groups of cultivated rice and wild rice accessions including *O. sativa* tropical *japonica*, *O. sativa aromatic*, *O. sativa indica*, *O. sativa aus* and *O. rufipogon*. Therefore, we conclude that *OsIRO3* may be a novel functional gene associated with alkalinity tolerance in temperate *japonica*.

## Conclusion

In the present study, 295 *japonica* rice varieties were collected to evaluate the tolerance of alkalinity stress at the seedling stage. Eight QTLs significantly related to the SAT, SNC and SNK were identified by GWAS. A common QTL on chromosome 3 associated with the SAT, SNC and SNK was selected for further analysis. Based on sequence analysis, *LOC_Os03g26210* maybe a novel functional gene for alkalinity tolerance of *japonica* rice. This study provides resources for improving rice alkalinity-tolerant breeding.

## Methods

### Plant material and genotyping

The natural population comprised 295 *japonica* rice varieties, which were collected from the Heilongjiang, Jilin and Liaoning provinces in China and other countries including Japan, the Republic of Korea, the Democratic People’s Republic of Korea and Russia (Additional file 2: Table S2). All 295 *japonica* rice varieties were belong to temperate *japonica* rice. Most of the varieties in this population were selected from a previous study (Zheng et al. 2015).

Total DNA was extracted from leaves of each variety using an *EsayPure* Plant Genomic Kit (TransGen Biotech, Beijing, China). The DNA was physically sheared into ~ 500 bp fragments using a Covaris S2 (Covaris). The fragmented DNA was used for DNA library construction with an NEBNext DNA Library Prep Reagent Set for Illumina (BioLabs). The DNA library was sequenced using an Illumina HiSeq XTen (Illumina Co, Ltd.) at the Beijing Genomics Institute (BGI). All reads were mapped against Nipponbare-Reference-IRGSP-1.0 pseudomolecules using BWA software (Li and Durbin 2009). SNP calling for each sample were performed using HaplotypeCaller in GATK software based on GATK Best Practices. The number of SNPs with an MAF ≥ 5% and a missing rate ≤ 20% ultimately identified in this study was 788,396. The chromosomal distribution of the SNPs is shown in Additional file 9: Figure S3.

### Alkalinity tolerance evaluation at the seedling stage

Alkalinity tolerance was evaluated in a hydroponics system at Northeast Agricultural University’s experimental station in 2018. The experiment followed a randomized complete block design with two treatments (control and alkalinity treatment) and three replications of each treatment. Fifty seeds of each accession were naturally air dried and kept at 55 °C for 5 days to break dormancy. Then, the seeds were surface sterilized with 70% ethyl alcohol and washed three times with sterile water. Next, the seeds were soaked in water for approximately 2 days and allowed to germinate for 1 day. Finally, thirty-six uniform germinated seeds of each variety were sown in 96-well plates supported by a plastic container containing Yoshida’s culture solution (Yoshida et al. 1976) and grown to the two leaf stage. The seedlings were grown in a phytotron glasshouse maintained at 14 h light/10 h dark photoperiod (27 °C/25 °C) and relative humidity of 70%. For the alkalinity treatment, the culture solution was replaced by a fresh solution containing 0.15% Na_2_CO_3_ for 7 days, and the seedlings were then transferred to Yoshida’s culture solution and allowed to continue growth. After 5 days of recovery growth, the seedlings were assessed to determine their SAT on a 1–9 scale. For scores of 1, 2, 3, 4, 5, 6, 7, 8, and 9, the percentage of dry and yellow leaves was ≤20, between 21 and 30, between 31 and 40, between 41 and 50, between 51 and 60, between 61 and 70, between 71 and 80, between 81 and 90, and > 90, respectively. Then, the shoots of each sample were harvested. The samples were oven dried at 105 °C for 20 min and 60 °C for 1 week prior to measuring the ion content of the shoots. A total of 0.1 g of dried sample was ground and then digested with 0.1 N Nitric acid (Fisher Scientific) at 70 °C for 8 h (Campbell et al. 2017). The SNC and SKC were analysed using a flame photometer (*Sherwood410*, Cambridge, UK) (Li et al. 2017).

### Population genetic analysis

An NJ tree was constructed using MEGA7 software (Saitou and Nei 1987; Kumar et al. 2016). The format of the exported phylogenetic tree (Newick format) was modified using the online tool Interactive Tree of Life (iTOL, https://itol.embl.de/) (Letunic and Bork 2011). GCTA software was used to conduct a PCA to estimate the number of subpopulations (Yang et al. 2011). ADMIXTURE software was used to calculate the genetic component for each variety (H Alexander et al. 2009), and each variety was assigned to a subpopulation for which the membership value (Q value) was > 0.65 (zheng et al. 2015). LD was calculated using PopLDdecay software (Zhang et al. 2018). The Pairwise *r*^2^ was calculated for all SNPs in a 50-kb window and averaged across the whole genome and 12 chromosomes separately. The LD decay was measured as the chromosomal distance at which the average pairwise correlation coefficient (*r*^2^) decreased to half its maximum value.

### GWAS analysis

GWAS was conducted via the MLM method using Tassel 5.0 software (Bradbury et al. 2007). The population structure (Q) and kinship calculated among individuals were used to adjust the population structure. The threshold was set at *P* = 6.34E-08 (that is 0.05/788,396) by the Bonferroni correction method. To obtain the loci with the lowest *P* value, redundant SNP were filtered in an LD interval, and the SNP with the minimum P value was considered the lead SNP. The manhattan plot and quantile-quantile (Q-Q) plot were produced by the CMplot package in R.

### RNA extraction and quantitative real-time PCR analysis

For analysis of candidate gene expression in leaves, ten each of the alkalinity-tolerant and alkalinity-sensitive rice accessions were selected according to the SAT of 295 *japonica* rice accessions. The procedure and management of the experiment were the same as those in the above-mentioned experiment. After 48 h of alkalinity stress implemented with 0.15% of Na_2_CO_3_, five shoots from each variety were sampled under alkaline and normal conditions. Total RNA was extracted from rice shoots using a *TranZol* Up RNA kit (TransGen Biotech). All samples were treated with DNase I (TransGen Biotech). Complementary DNA was synthesized from total RNA using HiFiScript cDNA Synthesis Kit (CoWin Biosciences, Beijing, China). qRT-PCR was performed using 2 × Fast qPCR Master Mixture (DINING, Beijing, China) on Analytik Jena qTOWER system (German). Additional file 8: Table S6 summarizes the gene accessions and primers used for qRT-PCR in this study. The mRNA level of these genes was determined with the housekeeping gene *Actin1* (Li et al. 2018) as an internal control. Relative gene expression levels were determined using the 2 ^−ΔΔCt^ method (Livak and Schmittgen 2001). The data shown in figures and tables are mean values of three replicates.

### Candidate gene prediction, sequencing, and sequence alignment

According to LD decay analysis of chromosome 3, a 325-kb reference sequence of the mapped QTL regions was identified as the candidate region. Based on the gene annotation and RNA sequencing data collected under alkalinity stress and control condition in a previous study (Li et al. 2018), the genes related to stress were selected as candidate genes. GO enrichment analysis of candidate genes was performed by using agriGO (Tian et al. 2017). Then, the corresponding candidate genes were cloned by PCR and sequenced in ten alkalinity-tolerant and ten alkalinity-sensitive rice varieties. Sequence alignment was performed in DNAMAN software with the genes in the Nipponbare genome as a reference.

### Validation of candidate genes with molecular markers

A 7-bp indel difference between the alkalinity-tolerant and alkalinity-sensitive rice cultivars for the candidate gene *LOC_Os03g26210* was designed as an indel maker using the conserved flanking sequences as primers (forward primer: CCCTTGCTGTTATTGCTC; reverse primer: GAGGAGGGCGAAGATTGA). The marker was further used to genotype 126 *japonica* rice varieties by PCR with the following PCR protocol: 30 cycles at 94 °C for 30 s, 55 °C for 30 s, and 72 °C for 1 min. PCR products were separated on 6% denaturing polyacrylamide gels and stained with AgNO_3_.

## Additional files

Additional file 1: **Table S1**. Phenotypic variation in 295 *japonica* rice varieties under alkalinity condition. (DOCX 13 kb)

Additional file 2: **Table S2.** Geographical distribution of 295 japonica rice varieties (XLSX 22 kb)

Additional file 3 : **Figure S1.** LD decay analysis of the whole genome in 295 *japonica* rice varieties. (TIFF 534 kb)

Additional file 4: **Figure S2**. LD decay analysis of 12 chromosomes in 295 *japonica* rice varieties. The x-axis represents the distance (bp) of the SNP; the y-axis represents r^2^. (TIFF 708 kb)

Additional file 5: **Table S3.** Summary of functional annotation of the genes in the candidate region on chromosome 3 (XLSX 12 kb)

Additional file 6: **Table S4.** GO enrichment analysis of the genes in candidate region. (XLSX 15 kb)

Additional file 7: **Table S5.** RNA-seq dates of the genes in candidate region. (XLSX 12 kb)

Additional file 8: **Table S6.** Primers for qRT-PCR in this study. (DOCX 14 kb)

Additional file 9: **Figure S3**. The chromosomal distribution of the SNPs used for GWAS in this study. (JPG 633 kb)

## Abbreviations

GWAS: Genome-wide association study
InDel: Insertion-deletion
LD: Linkage disequilibrium
MAF: Minor allele frequency
QTL: Quantitative trait locus
SAT: the score of alkalinity tolerance
SKC: Concentration of K^+^ in the shoots
SNC: Concentration of Na^+^ in the shoots
SNK: Na^+^/K^+^ ratio of shoots
SNP: Single-nucleotide polymorphism
