# Supplementary material for: Genome-wide association study and candidate gene analysis of alkalinity tolerance in japonica rice germplasm at the seedling stage
Source: Rice (N Y). 2019 Apr 11;12:24. doi: 10.1186/s12284-019-0285-y (PMC6459459; doi:10.1186/s12284-019-0285-y)
Supplement: Supplementary file 1 — Table S1. Phenotypic variation in 295 japonica rice varieties under alkalinity condition. (DOCX 13 kb) [file 12284_2019_285_MOESM1_ESM.docx]

**Table S1**. Phenotypic variation in 295 *japonica* rice varieties under alkalinity condition.

| Trait | Mean | SD | Range | Skewness | Kurtosis | CV (%) |
| --- | --- | --- | --- | --- | --- | --- |
| SAT | 5.02 | 2.28 | 1-9 | 0.27 | -0.90 | 45.50 |
| SNC (mmol/ml) | 5.26 | 1.85 | 1.30-14.57 | 0.91 | 1.18 | 35.20 |
| SKC (mmol/ml) | 2.67 | 0.74 | 1.15-4.74 | 0.44 | -0.26 | 27.82 |
| SNK | 2.02 | 0.63 | 0.64-5.17 | 0.61 | 1.53 | 31.08 |

CV: Indicates the coefficient of variation.
