# Supplementary figures and images for: Genome-wide association study and candidate gene analysis of alkalinity tolerance in japonica rice germplasm at the seedling stage
Source: Rice (N Y). 2019 Apr 11;12:24. doi: 10.1186/s12284-019-0285-y (PMC6459459; doi:10.1186/s12284-019-0285-y)

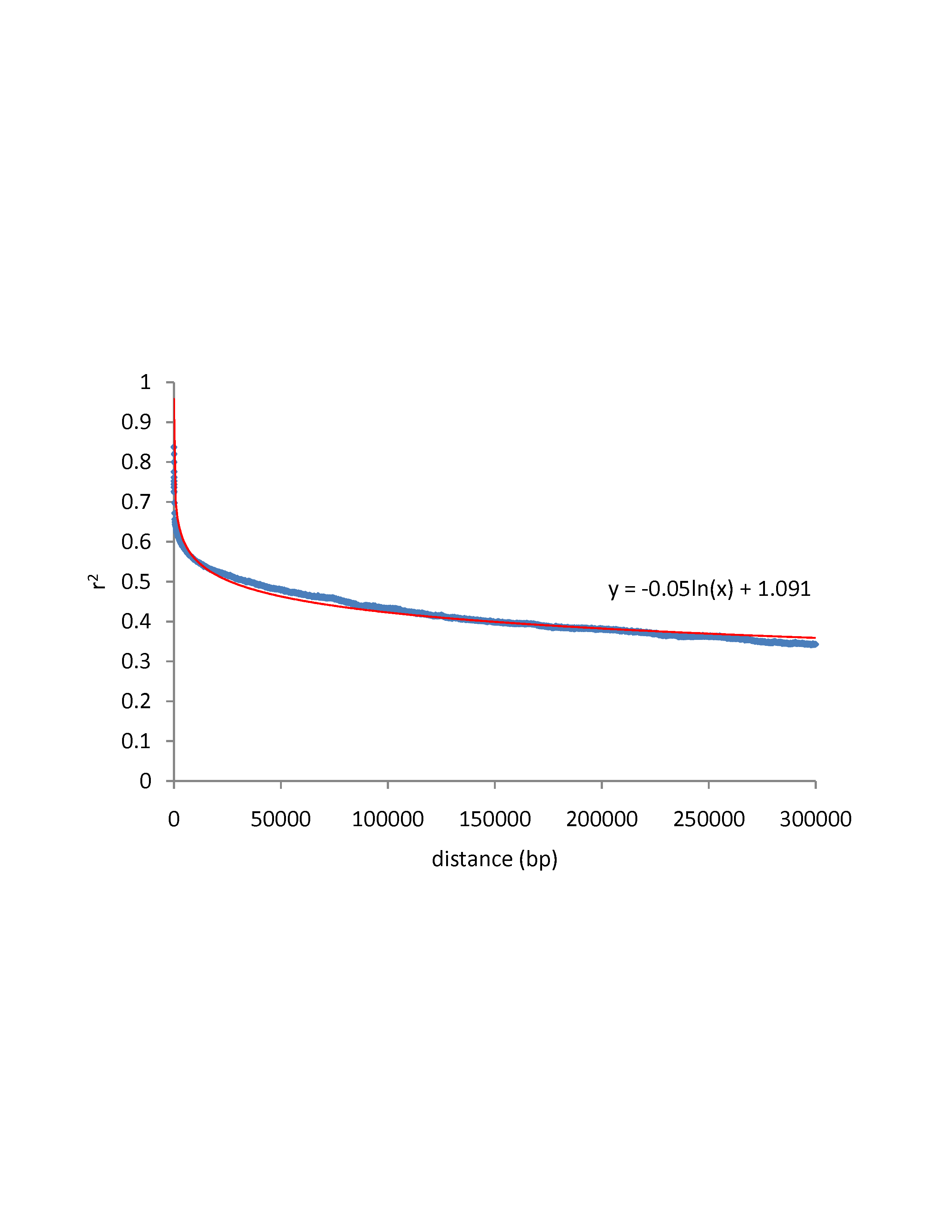

Supplement: Supplementary file 3 — : Figure S1. LD decay analysis of the whole genome in 295 japonica rice varieties. (TIFF 534 kb) [file 12284_2019_285_MOESM3_ESM.tiff]

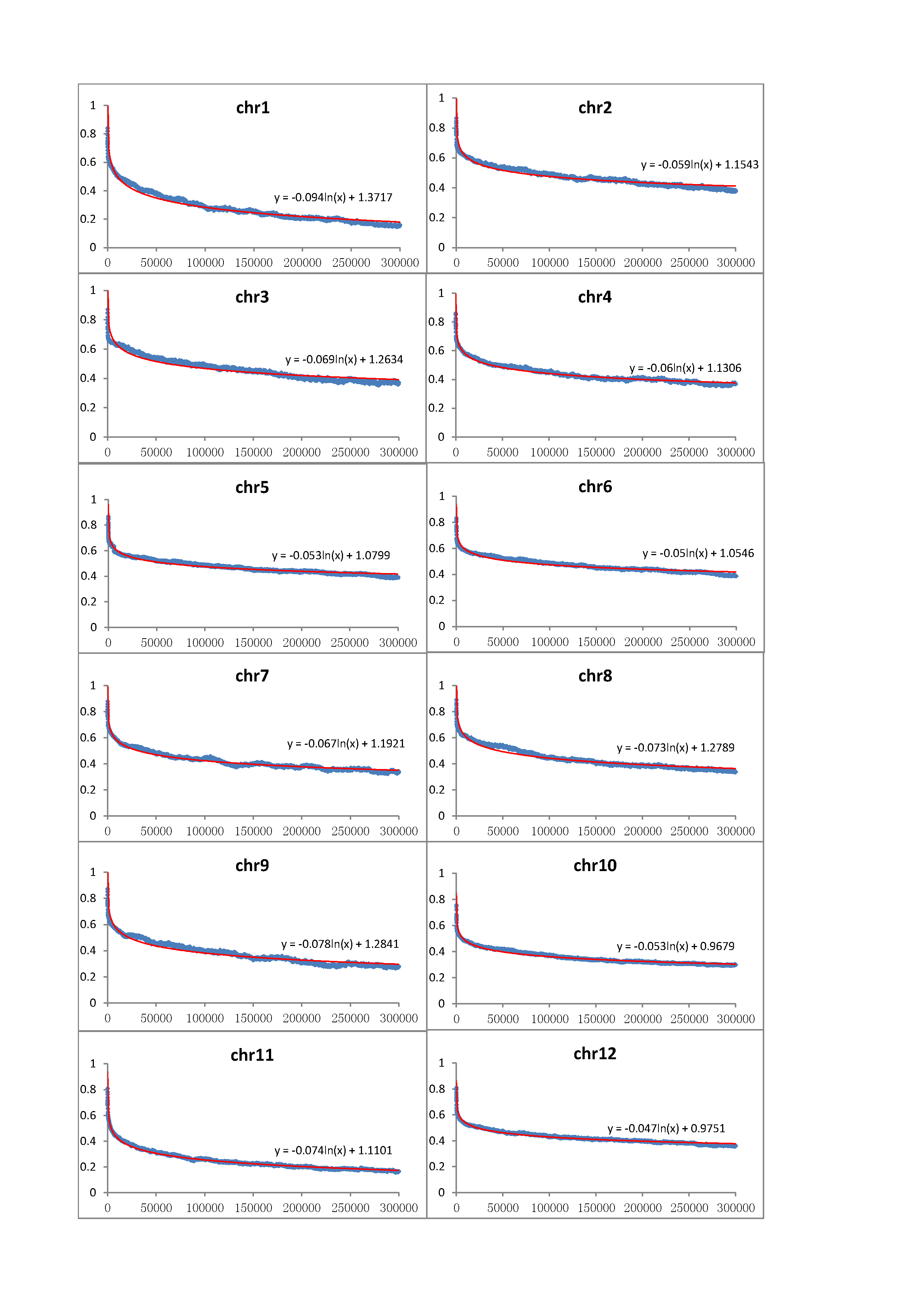

Supplement: Supplementary file 4 — Figure S2. LD decay analysis of 12 chromosomes in 295 japonica rice varieties. The x-axis represents the distance (bp) of the SNP; the y-axis represents r2. (TIFF 708 kb) [file 12284_2019_285_MOESM4_ESM.tiff]

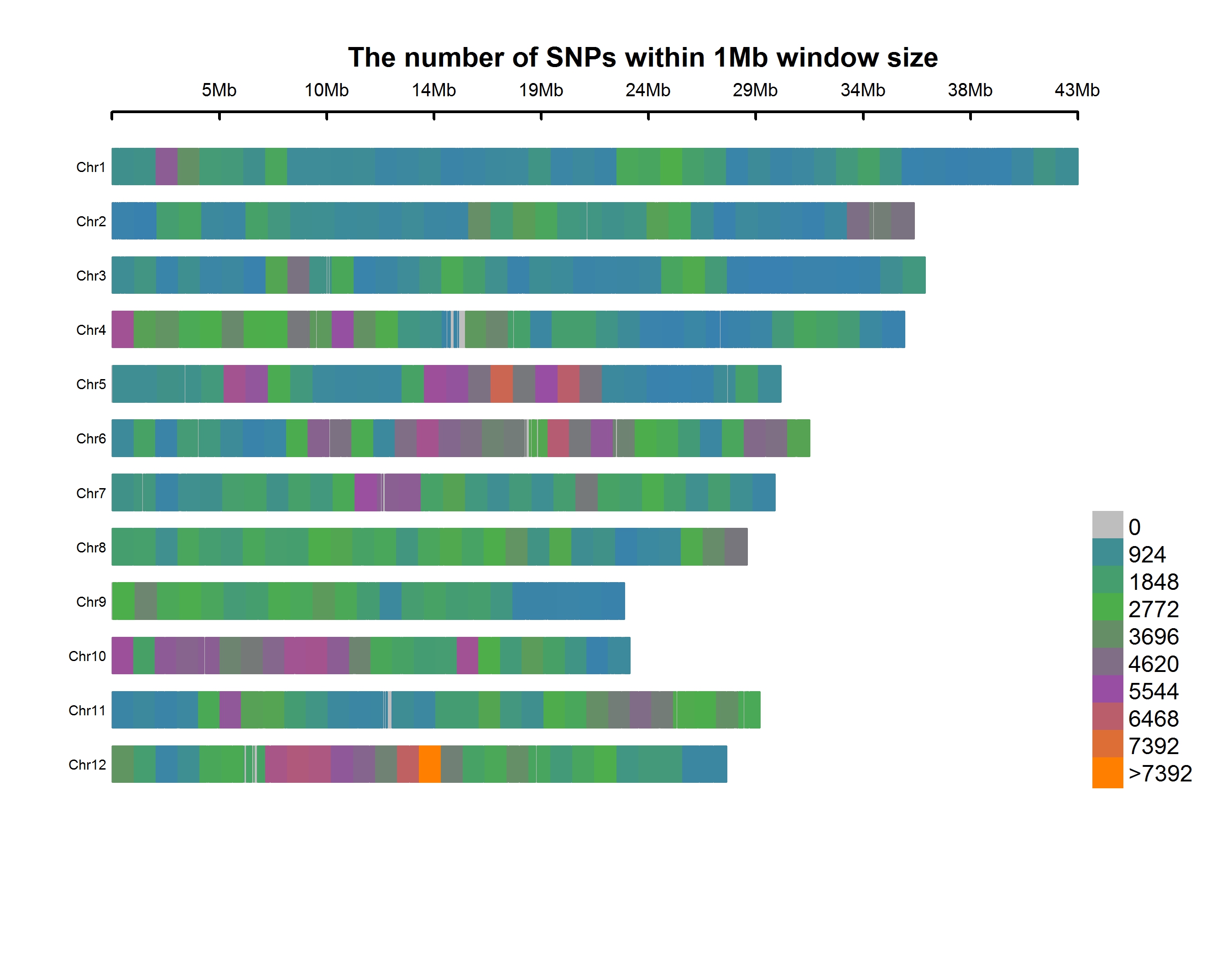

Supplement: Supplementary file 9 — Figure S3. The chromosomal distribution of the SNPs used for GWAS in this study. (JPG 633 kb) [file 12284_2019_285_MOESM9_ESM.jpg]
