# Supplementary material for: Genome-wide association study and candidate gene analysis of alkalinity tolerance in japonica rice germplasm at the seedling stage
Source: Rice (N Y). 2019 Apr 11;12:24. doi: 10.1186/s12284-019-0285-y (PMC6459459; doi:10.1186/s12284-019-0285-y)
Supplement: Supplementary file 8 — Table S6. Primers for qRT-PCR in this study. (DOCX 14 kb) [file 12284_2019_285_MOESM8_ESM.docx]

| Gene accession | Forward primers (5'-3') | Reverse primers (5'-3') |
| --- | --- | --- |
| *LOC_Os03g25920* | TATTGTGATGAGGAGGAGTGA | CGAGGCTGTAGAGGAAGTT |
| *LOC_Os03g25940* | GGTAGTGATAGCCCTTGTTAG | GACTTGGCAGACCAGGAT |
| *LOC_Os03g26080* | GCAACTGGTAGATCAGCCCA | CTTGCCAAGCAAAGTGCTCC |
| *LOC_Os03g26090* | AGTTGACACACTCTCTGCCTC | ACTGAGCTAAAGCAGCAATCA |
| *LOC_Os03g26130* | TCCGGCCAGGAAAGGATTGTC | TCGTCGCACGCGAGGAGCAT |
| *LOC_Os03g26210* | TGGTCGATTGGTTTTCAGCAG | AACCTTCCTCGGGACCTTCT |
| *LOC_Os03g26229* | CCGCTATGGTCTCCTTGGTG | GAATGGATTTCACCGTGCCG |
| *LOC_Os03g26260* | TGGGCATACCTCAGTGGTTTC | CGCATTGGTACTTGCGTTGT |
| *LOC_Os03g26300* | GGTTCCGTTCCAACTCCGA | GCAGGGAGGTGGAGATAATCA |
| *LOC_Os03g26370* | CAGCCGAGCAGGAGGATTAT | ACCACTCGCAACTCCAACG |
| *LOC_Os03g26430* | TGCGTTCCACACGTACAAGA | CGACCAGAACACCTGGTTGA |
| *LOC_Os03g26450* | GAATCGCTCTGCTCCAAT | GGTATAGCTGCAACTATCACTA |
| *Actin1* | TGGCATCTCTCAGCACATTCC | TGCACAATGGATGGGTCAGA |

Table S5 Primers for qRT-PCR in this study
